# Computational functional annotation and structural modelling of hypothetical proteins in Stenotrophomonas maltophilia NCTC10958

**DOI:** 10.1099/acmi.0.001195.v3

**Published:** 2026-11-05

**Authors:** Iman Hussein Fadhiladeen Alnaqshabandy

**Affiliations:** 1 Department of Scientific Research Center, College of Science, University of Duhok, Duhok, Kurdistan Region, Iraq

**Keywords:** antimicrobial resistance, hypothetical proteins, *Stenotrophomonas maltophilia*, virulence factors

## Abstract

Antimicrobial resistance represents a significant global health challenge, specifically concerning the escalation of multidrug-resistant Gram-negative bacteria. *Stenotrophomonas maltophilia* has emerged as a prevalent pathogen affecting immunocompromised patients in intensive care units. Its incidence has notably increased, particularly during the coronavirus disease 2019 pandemic, complicated by resistance mechanisms such as enzymatic degradation and biofilm formation. Despite its clinical importance, the functional roles of many genomic regions remain obscure. This study aimed to characterize 61 hypothetical proteins from *S. maltophilia* NCTC10958 to elucidate their involvement in virulence and adaptation. Functional annotation revealed that a significant proportion of these proteins are membrane-associated enzymes and transporters essential for environmental adaptation. Key findings include the identification of putative virulence factors involved in biofilm formation and motility, such as phosphoglycerate mutase and type IV pilus assembly proteins. Crucially, the analysis uncovered key functional determinants, including a ComEC/Rec2 family competence protein involved in DNA uptake and a mycobacterial membrane protein large family transporter linked to cell envelope processes and transport. Structural modelling validated high-confidence tertiary structures for these targets, confirming their biological feasibility. These insights provide a foundation for understanding the pathogenicity of *S. maltophilia* NCTC10958 and highlight specific HPs as promising candidates for therapeutic intervention.

## Data Summary

All data generated or analysed during this study are included in this published article. The complete genome sequence of *Stenotrophomonas maltophilia* strain NCTC10958 analysed in this study was retrieved from the National Center for Biotechnology Information (NCBI) database under GenBank Assembly ID GCA_900475405.1 (RefSeq: NZ_LS483377.1). The specific hypothetical protein sequences analysed are derived from this genomic dataset. A previous version of this manuscript was deposited as a preprint on Research Square (https://doi.org/10.21203/rs.3.rs-7217113/v1).

## Introduction

*Stenotrophomonas maltophilia* is a globally emerging multidrug-resistant (MDR) pathogen and a leading cause of nosocomial infections, particularly among immunocompromised individuals in intensive care units [1–8]. The treatment of *S. maltophilia* infections is increasingly challenging due to its intrinsic resistance mechanisms, which include the expression of efflux pumps, modification of antibiotic target sites and the production of hydrolytic enzymes [3, 9, 10]. Furthermore, the pathogen’s ability to form biofilms on both biotic and abiotic surfaces significantly enhances its persistence and resistance to host immune defences and antimicrobial agents [11–14]. Micro-organisms within these biofilms exhibit increased genetic adaptability and resistance to host immune defences, often tolerating antibiotic concentrations far higher than their planktonic counterparts [15]. This adaptive capability has been particularly concerning during the severe acute respiratory syndrome coronavirus 2 pandemic, where increased antibiotic usage in hospitalized coronavirus disease 2019 patients has correlated with a rise in secondary *S. maltophilia* infections [14, 16].

Developing novel therapeutic strategies requires a comprehensive understanding of the biological mechanisms driving *S. maltophilia* pathogenicity. While known virulence factors such as hydrolytic enzymes, flagella, pili and quorum sensing systems have been characterized [8, 11], a significant portion of the *S. maltophilia* genome remains functionally undefined. These uncharacterized regions, annotated as ‘hypothetical proteins’ (HPs), represent a major knowledge gap. HPs may harbour critical determinants for bacterial metabolism, environmental adaptation or novel resistance mechanisms that have yet to be exploited for drug discovery.

*In silico* approaches have proven highly effective in bridging this gap. Computational functional annotation has successfully identified essential proteins and drug targets in various pathogens, including *Mycobacterium abscessus* [17], *Klebsiella pneumoniae* [18] and *Enterobacter cloacae* [19]. However, the specific genomic underpinnings of *S. maltophilia* strain NCTC10958 remain under-explored. Currently, over 500 proteins in this clinically relevant strain are designated as hypothetical, obscuring potential insights into its specific adaptations to the hospital environment. While automated resources such as the AlphaFold Protein Structure Database (AFDB) and the 3D Protein Domain Database (TED) provide predicted 3D coordinates across public proteomes, automated entries for HPs in UniProt often remain annotated without functional context. Unlike automated global domain assignments (such as AFDB/TED), an integrated, multi-tool consensus approach combining domain architecture with subcellular localization, signal peptide screening and physicochemical profiling is required to link uncharacterized proteins directly to specific microbial phenotypes and disease mechanisms.

In this study, we aimed to functionally characterize the HPs of *S. maltophilia* strain NCTC10958 using an integrative in silico pipeline. By analysing subcellular localization, Gene Ontology (GO), physicochemical properties and 3D structures, we seek to define the roles of these proteins in bacterial pathogenesis and adaptation mechanisms (genomic plasticity, stress responses and cell envelope transport). Ultimately, resolving the functions of these HPs may unveil novel virulence and adaptation factors and facilitate the discovery of alternative therapeutic targets to combat this persistent MDR pathogen.

## Methods

### Sequence retrieval and dataset preparation

The complete genome sequence of *S. maltophilia* strain NCTC10958 was retrieved from the National Center for Biotechnology Information (NCBI) database (GenBank Assembly ID: GCA_900475405.1; RefSeq: NZ_LS483377.1). This genome consists of 4,481,118 bp with a G+C content of 66.5 mol% and contains 4,106 predicted genes. Coding sequences (CDSs) annotated specifically with terms including ‘HP’, ‘uncharacterized protein’ or ‘protein of unknown function’ were systematically analysed in FASTA format. From this dataset, a total of 561 CDSs annotated as HPs were extracted in FASTA format for downstream analysis. The list of HPs collected with their required information, which are used for further analysis, is shown in Table S1 (available in the online Supplementary Material).

### Functional annotation pipeline

To ensure high-confidence predictions and minimize false positives, a multi-tier consensus-based functional annotation strategy was employed across seven independent domain-prediction databases. The sequences were analysed using a suite of bioinformatics tools to identify conserved domains, motifs and superfamilies (Table S2). Conserved Domain Architecture Retrieval Tool (CDART) [20] and Conserved Domain Database (CDD)-Basic Local Alignment Search Tool (BLAST) (*E*-value threshold ≤10^−3^) [21–23] were used to detect conserved domain architectures. InterPro [24] was utilized to integrate predictive models from multiple member databases. CATH and SUPERFAMILY [25, 26] were applied for structural classification and evolutionary lineage prediction. ScanProsite [27] and SMART [28, 29] were used to identify functional motifs and domain signatures. All tools were executed with default parameters. Subsequently, 61 HPs were selected for further analysis as they displayed functional domains or motifs in a minimum of three of the bioinformatics tools and followed a schematic diagram as shown in Fig. S1. An HP was selected for further detailed characterization only if a functional domain or motif was predicted concordantly by at least three independent tools out of seven. Out of the 561 initial HPs, 500 failed to meet this strict consensus cutoff or yielded uninformative hits of unknown function and were excluded. This filtering process yielded a final curated dataset of 61 high-confidence targets. GO terms were assigned using the Annotation Retrieval of Gene Ontology Terms (ARGOT) server [30] to classify proteins by molecular function, biological process and cellular component.

### Physicochemical and subcellular localization profiling

Physicochemical parameters, including molecular weight, isoelectric point (pI), instability index and grand average of hydropathicity (GRAVY), were calculated using ExPASy ProtParam [31]. Subcellular localization was predicted using a combinatorial approach with CELLO v.2.5 [32, 33] and PSORTb v.3.0 [34] to distinguish between cytoplasmic, periplasmic, membrane and extracellular proteins. This profiling serves to prioritize high-value candidates. Because virulence factors and efflux machinery typically function at the bacterial surface, mapping localization identifies targets directly involved in host interactions and adaptation. The presence of N-terminal signal peptides and cleavage sites was determined using SignalP [35].

### Tertiary structure prediction

3D structural models were generated to provide mechanistic insights. Although automated monomeric models are available via AFDB, template-based homology modelling was conducted to independently cross-validate structural fidelity against experimentally solved template structures with known catalytic domains and ligand-binding pockets. SWISS-MODEL [36] was employed as the primary tool due to its integrated homology modelling capabilities, which facilitate functional inference from conserved templates. Template selection was guided by Protein Basic Local Alignment Search Tool (BLASTP) sequence alignment to ensure high sequence identity (>30%) and maximal coverage. Model quality was rigorously assessed using Global Model Quality Estimation (GMQE) scores and Qualitative Model Energy Analysis values to ensure structural reliability. Additionally, for candidate membrane-associated proteins [such as mycobacterial membrane protein large (MmpL) and major facilitator superfamily (MFS) transporters], predicted structures were analysed for membrane-embedding topology to confirm active-site accessibility.

## Results and discussions

### Functional classification and GO

To characterize the 61 selected HPs from *S. maltophilia* NCTC10958, we employed a consensus-based annotation pipeline. Functional classification revealed a diverse array of roles: 22 proteins were classified as binding proteins, 28 as enzymes, 6 as transport proteins and 4 as regulatory proteins (Table S3, Fig. S2). GO analysis using ARGOT further categorized 78.7% (48/61) of these proteins. In the cellular component category, the majority were associated with integral membrane components (50%) and membranes (34%), consistent with the high proportion of transport and signalling proteins identified.

Biological process analysis highlighted ‘regulation of transcription’ (GO:0006355; 12.5%) and ‘proteolysis’ (GO:0006508; 10%) as dominant categories. This suggests that a significant portion of the uncharacterized genome in this strain is dedicated to regulatory adaptation and protein quality control, which are critical for survival in fluctuating hospital environments. Molecular function analysis showed a prevalence of ATP binding (6.5%), transferase activity (6.5%) and kinase activity (6.5%), indicating active metabolic engagement (Table S5, Fig. 1a).

**Fig. 1.**
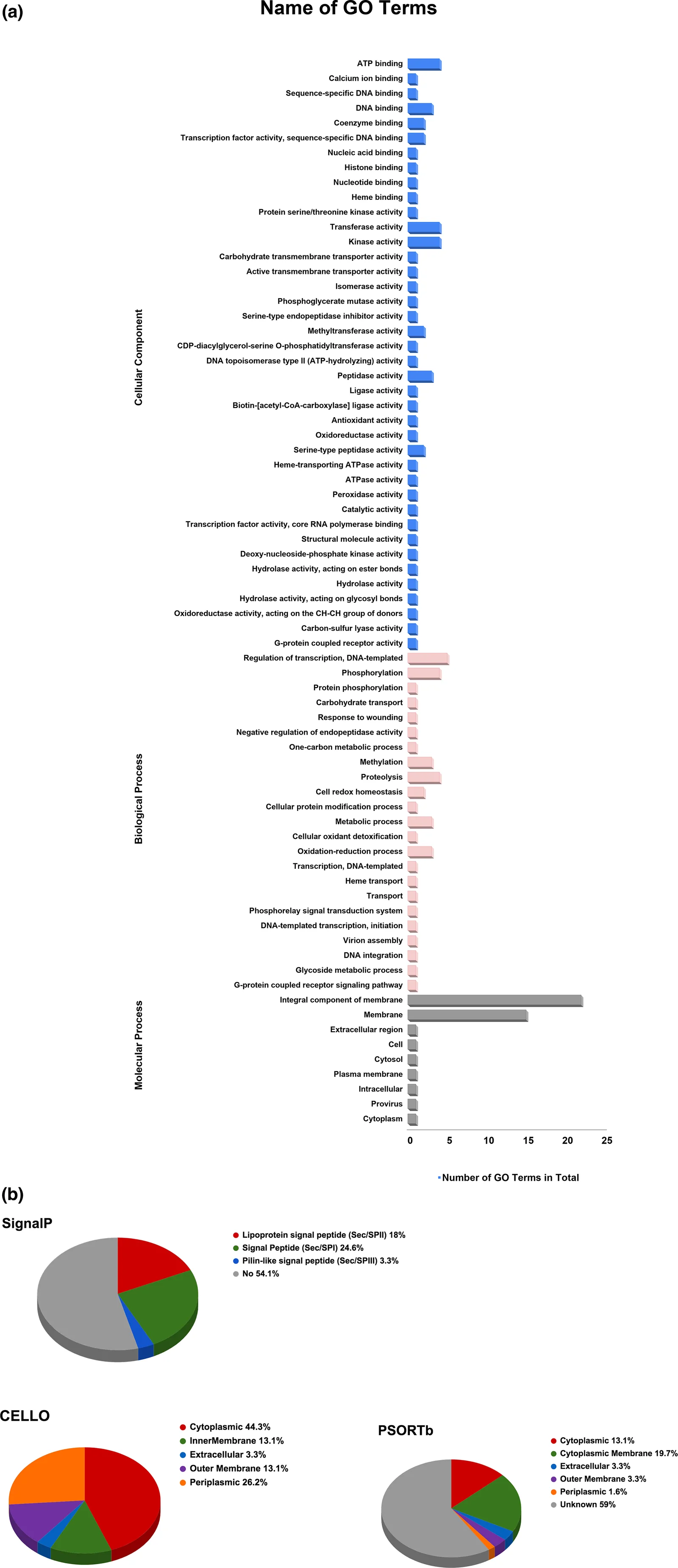
Subcellular localization and functional classification of the 61 HPs. (**a**) GO classification. Bar chart representing the frequency of GO terms across three categories: molecular function (blue), biological process (pink) and cellular component (grey). (**b**) Predicted localization and secretion signals. Charts display the subcellular distribution predicted by CELLO and PSORTb, alongside SignalP analysis identifying standard (24.6%), lipoprotein (18%) and pilin-like (3.3%) signal peptides.

### Physicochemical properties and subcellular localization

Physicochemical profiling provided insights into the stability and solubility of the HPs (Fig. S3a). The majority (64%) were neutral (pI 5–9), while 36% were eccentric (7 acidic, 15 basic). GRAVY indices revealed that 40 proteins (65.5%) were hydrophilic, suggesting cytoplasmic or periplasmic localization, while 21 (34%) were hydrophobic, consistent with membrane-embedded functions (Table S6, Fig. 3Sb).

Subcellular localization predictions using CELLO and PSORTb yielded complementary data. CELLO predicted a predominant cytoplasmic distribution (44.3%) followed by periplasmic (26.2%) and outer membrane (13.1%) locations. PSORTb, while more conservative (returning 59% as ‘unknown’), positively identified 19.7% as cytoplasmic membrane proteins. SignalP analysis identified signal peptides in 24.6% of the HPs, including specific subsets predicted as lipoprotein signal peptides (18%) and pilin-like signal peptides (3.3%) (Table S4, Fig. 1b). These secretion signals indicate potential extracellular or envelope-associated localization, which may participate in general physiological processes, cell envelope integrity or structural maintenance, as well as potential interactions with the external host environment.

### Virulence factors: adhesion, biofilm and motility

A primary aim of this study was to identify factors contributing to the persistence of *S. maltophilia*. Our analysis revealed several proteins with high homology to known virulence determinants (Table 1). We identified WP_005409484.1 as a putative phosphoglycerate mutase. While primarily a metabolic enzyme, this protein has been implicated in mediating early-stage attachment to abiotic surfaces [37]. Its identification in *S. maltophilia* NCTC10958 suggests a potential role in biofilm initiation, distinct from mature biofilm maintenance [8].

**Table 1.** Key HPs annotated as potential virulence factors and resistance determinants in *S. maltophilia* NCTC10958

No.	Accession no.	Predicted function	Biological role/significance
	**Virulence factors (adhesion and biofilm**)		
**1**	WP_005409484.1	Phosphoglycerate mutase	Putative involvement in early-stage biofilm attachment to abiotic surfaces
**2**	WP_005407536.1	Type IV pilus assembly protein	Essential for twitching motility, surface adherence and biofilm maturation
**3**	WP_005409996.1	Fimbrial protein (P-loop NTPase)	Fimbrial biogenesis; critical for host colonization
**4**	WP_012480646.1	Toxin coregulated pilus Q family	Homologous to *V. cholerae* virulence factors; colonization and toxin secretion
	**Efflux mechanisms and genomic adaptability**		
**1**	WP_111687834.1	MmpL family transporter	RND superfamily; likely involved in lipid secretion and cell envelope remodelling
**2**	WP_005408925.1	MFS transporter	MFS; efflux of antibiotics and toxins
**3**	WP_111687594.1	MFS transporter	MFS; efflux of antibiotics and toxins
**4**	WP_231914714.1	Heme ABC transporter	ATP-binding protein involved in nutrient uptake and toxin secretion
**5**	WP_145983607.1	ComEC/Rec2 family competence protein	Putative role in DNA uptake, natural transformation and horizontal gene transfer
	**Enzymatic defence and signalling**		
**1**	WP_111686702.1	Lipase (alpha/beta hydrolase)	Extracellular enzyme involved in lipid degradation and host tissue damage
**2**	WP_005408698.1	Clp protease	Chaperone involved in stress response and protein quality control
**3**	WP_005411207.1	Serine/threonine kinase (PASTA)	Regulation of cell wall homeostasis and *β*-lactam resistance
**4**	WP_111687316.1	EF-hand domain protein	Calcium-binding protein involved in signal transduction
	**Transcriptional regulation**		
**1**	WP_005409404.1	TetR/AcrR family regulator	Regulation of multidrug efflux pumps and osmotic stress response
**2**	WP_005408543.1	Arc-like DNA binding protein	Transcriptional regulator of aerobic/anaerobic metabolism

Several proteins involved in structural virulence determinants were also characterized. WP_005407536.1 was identified as a type IV pilus assembly protein. Type IV pili are critical for twitching motility, surface adherence and biofilm maturation, making them key virulence factors in *S. maltophilia*. Additionally, WP_005409996.1 was predicted as a fimbrial protein harbouring a P-loop nucleoside triphosphate hydrolase domain, and WP_012480646.1 was annotated as a toxin-coregulated pilus Q family protein. In *Vibrio cholerae*, this family is essential for colonization and toxin secretion [38], suggesting a similar pathogenic role in *S. maltophilia*.

### Efflux mechanisms and genomic adaptability

Multidrug resistance is a hallmark of *S. maltophilia*. Our analysis uncovered several novel putative resistance determinants (Table 1). WP_111687834.1 was annotated as a MmpL family transporter. MmpL proteins, belonging to the resistance–nodulation–division (RND) superfamily, are classically associated with lipid secretion and cell wall biosynthesis in *Mycobacterium* species. The presence of an MmpL homologue in *S. maltophilia* is a noteworthy finding, suggesting a potentially unique lipid-based secretion system or efflux mechanism essential for cell wall remodelling and envelope adaptation that differs from the well-characterized SmeABC pumps [39].

Furthermore, WP_145983607.1 was identified as a ComEC/Rec2 family competence protein. As an essential component of bacterial DNA uptake machinery, ComEC plays a critical role in natural transformation and horizontal gene transfer, contributing to the genomic adaptability and evolution of *S. maltophilia* [40]. Additionally, WP_005408925.1 and WP_111687594.1 were annotated as MFS transporters, which frequently mediate the efflux of antibiotics and toxins to support envelope adaptation and physiological survival under stress.

### Enzymatic defence and transcriptional regulation

Lytic enzymes play a dual role in nutrient acquisition and defence against host immune responses. We identified WP_111686702.1 as a lipase with an alpha/beta hydrolase fold, a domain known for diverse catalytic functions including biodegradation and pathogenicity [41]. Additionally, WP_005408698.1 and WP_111687296.1 were predicted as caseinolytic protease proteins (Table 1). These chaperones are essential for bacterial stress responses, degrading misfolded proteins to ensure survival under harsh conditions [42].

Transcriptional regulation is vital for adapting to the host environment. We identified three key regulators: WP_005407775.1 (CopG-like), WP_005408543.1 (Arc-like) and WP_005409404.1 (TetR/AcrR family). Rather than driving drug resistance, these regulators likely govern broader networks involved in stress responses, metal homeostasis and metabolic adaptation. The TetR family is particularly significant as it typically modulates cellular responses to osmotic and environmental stress [43], further highlighting the essential role of these uncharacterized regulators in *S. maltophilia* cellular adaptation.

### Metabolism and cell envelope integrity

Beyond direct virulence, several HPs were linked to cell envelope maintenance, a critical factor in resistance (Table 1). WP_005414259.1 was predicted as an epimerase, an enzyme vital for the polymerization of complex carbohydrates during cell wall synthesis [44]. Similarly, WP_111687805.1 was identified as the SecD subunit of the preprotein translocase complex, which utilizes the proton motive force to export virulence factors and membrane proteins.

We also noted the presence of kinase signalling pathways. WP_005411207.1 was identified as a Serine/threonine kinase with a penicillin-binding protein and serine/threonine kinase-associated (PASTA) domain. In other pathogens, PASTA kinases regulate cell wall homeostasis and *β*-lactam resistance [45], marking this protein as a high-value target for further investigation.

### Structural analysis

Tertiary structure prediction using SWISS-MODEL provided a high-confidence model for all 15 prioritized candidate HPs (Tables 1 and S8), which were selected based on integrated consensus across the full suite of bioinformatic prediction tools used in our functional annotation pipeline. To maintain visual clarity, six representative 3D structures exhibiting high model confidence and key topological features across core functional pathways are illustrated in Fig. 2, while the modelled structures for the remaining nine candidates are provided in Fig. S4. BLASTP sequence alignment was utilized to corroborate these predictions by confirming high conservation with the selected templates (Table S7). Model reliability was confirmed by high GMQE scores (range: 0.65–0.93, with the majority exceeding 0.80), high sequence coverage (71.4%–100%) and high sequence identity (>89%; Table S8). For example, the modelled structure of the MmpL transporter (WP_111687834.1; template A0A4Q1CS25.1.A, GMQE: 0.85, identity: 94.77%) revealed a classic transmembrane architecture consistent with RND pumps, while the ComEC/Rec2 competence protein (WP_145983607.1; template A0A699XY32.1.A, GMQE: 0.90, identity: 99.36%) exhibited transmembrane helical features characteristic of bacterial DNA translocation channels. The TetR/AcrR regulator (WP_005409404.1; template A0A4Z0DKD0.1.A, GMQE: 0.93, identity: 91.49%) demonstrated a characteristic helix-turn-helix DNA-binding domain. These structural insights, supported by sequence alignment, not only validate the functional annotations but also provide templates for future molecular docking studies to identify potential small-molecule inhibitors. It is important to emphasize that while automated resources such as AFDB and TED offer broad structural predictions across public proteomes, they frequently leave strain-specific HPs without functional context. By integrating multi-tool consensus domain mapping with subcellular localization, secretion signal profiling and structural topology assessments, our pipeline provides a functional framework for *S. maltophilia* NCTC10958 targets that remain uncharacterized in automated public databases.

**Fig. 2.**
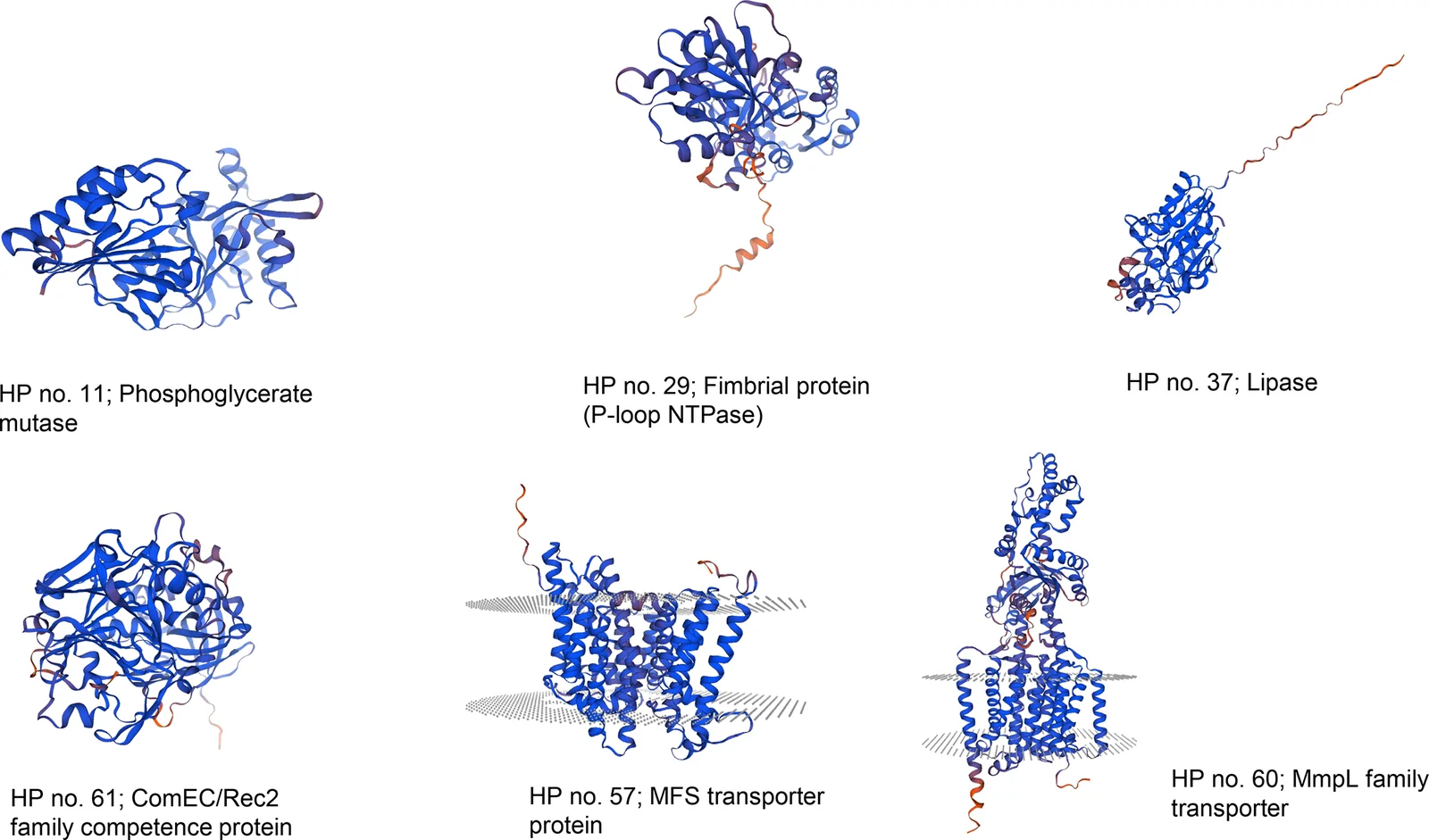
Predicted tertiary structures of six selected HPs. 3D structures were modelled using the SWISS-MODEL server via homology modelling. Templates were selected based on high sequence coverage, sequence identity (>70%) and high GMQE scores to ensure reliability. The modelled structures are rendered in blue, indicating high model confidence. Transmembrane segments are depicted embedded between two hypothetical lipid bilayer planes. The corresponding HP accession numbers are labelled adjacent to each structure.

## Limitations and future prospects

While this study provides significant insights into the functional landscape of *S. maltophilia* NCTC10958, several limitations must be acknowledged. First, the functional annotations and structural models presented here are based exclusively on computational predictions. However, as a hypothesis-generating study, these findings provide the necessary computational groundwork to prioritize specific targets for future experimental validation, thereby reducing the cost and time of wet-lab screening. Although we utilized high-confidence algorithms (SWISS-MODEL based on the AlphaFold v2 method) and consensus-based approaches to minimize errors, experimental validation through gene knockout, transcriptomic analysis or enzymatic assays is required to definitively confirm biological activity *in vivo*. Second, our analysis was restricted to a single strain (NCTC10958). Given the high genomic plasticity of *S. maltophilia*, the conservation of these HPs across diverse clinical and environmental isolates remains to be fully explored.

## Conclusion

This study functionally characterized 61 HPs in *S. maltophilia* NCTC10958, identifying overlooked resistance determinants including a ComEC/Rec2 family competence protein involved in DNA uptake and an MmpL family transporter. These proteins represent promising novel targets for therapeutic intervention against this MDR pathogen. Consequently, these computational insights provide a cost-effective framework to prioritize specific candidates for future experimental validation and drug discovery.

## Supplementary material

10.1099/acmi.0.001195.v3 Supplementary Material 1.

10.1099/acmi.0.001195.v3 Supplementary Material 2.

## Abbreviations

AFDB: AlphaFold Protein Structure Database
ARGOT: Annotation Retrieval of Gene Ontology Terms
blast: Basic Local Alignment Search Tool
BLASTP: Protein Basic Local Alignment Search Tool
CDS: coding sequence
GMQE: Global Model Quality Estimation
GO: Gene Ontology
GRAVY: grand average of hydropathicity
HP: hypothetical protein
MDR: multidrug-resistant
MFS: major facilitator superfamily
MmpL: mycobacterial membrane protein large
PASTA: penicillin-binding protein and serine/threonine kinase-associated
pI: isoelectric point
RND: resistance–nodulation–division
TED: 3D Protein Domain Database
