# Supplementary material for: Computational functional annotation and structural modelling of hypothetical proteins in Stenotrophomonas maltophilia NCTC10958
Source: Access Microbiol. 2026 Nov 5;8(11):001195.v3. doi: 10.1099/acmi.0.001195.v3 (PMC13752025; doi:10.1099/acmi.0.001195.v3)
Supplement: Supplementary file 1 [file acmi-8-01195-s001.pdf]

# Computational Functional Annotation and Structural Modeling of Hypothetical Proteins in *Stenotrophomonas maltophilia* NCTC10958

**Author: Iman Hussein Fadhiladeen Alnaqshabandy[1*]**

**Affiliation:** [1] Department of Scientific Research Center, College of Science, University of Duhok, Duhok, Kurdistan Region, Iraq

***Corresponding Author:** Iman Hussein Fadhiladeen Alnaqshabandy

**Email:** iman.fadhiladeen@uod.ac

**ORCID: 0009-0006-9550-363X**

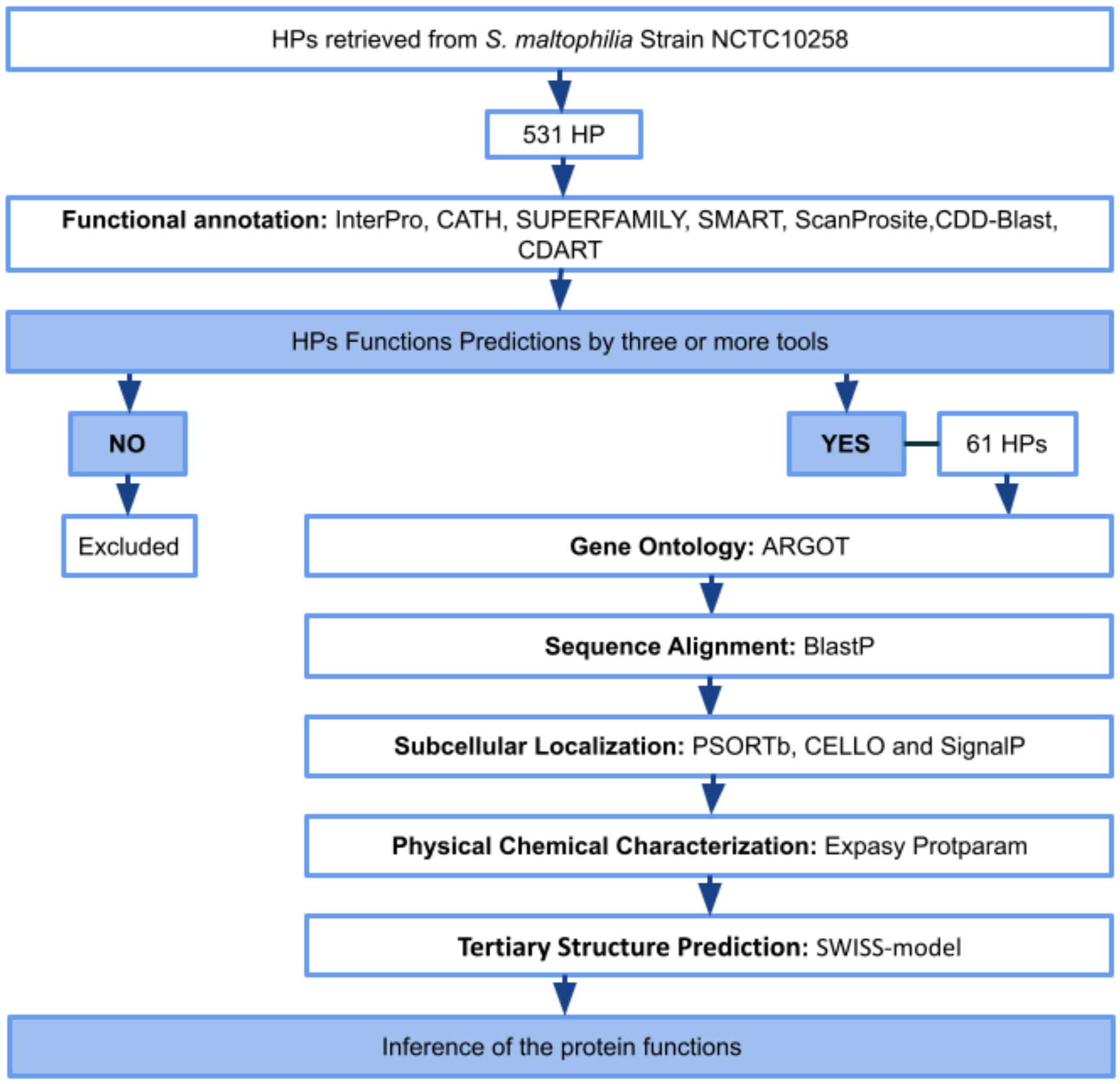


**Figure S1** Workflow used for the annotations of HPs in this study, the diagram shows the steps followed to predict the functions of the 531 HPs in *S. maltophilia* strain NCTC10258. The filled bar with blue color represents the predicted protein functions that are shown in at least three different bioinformatics tools.

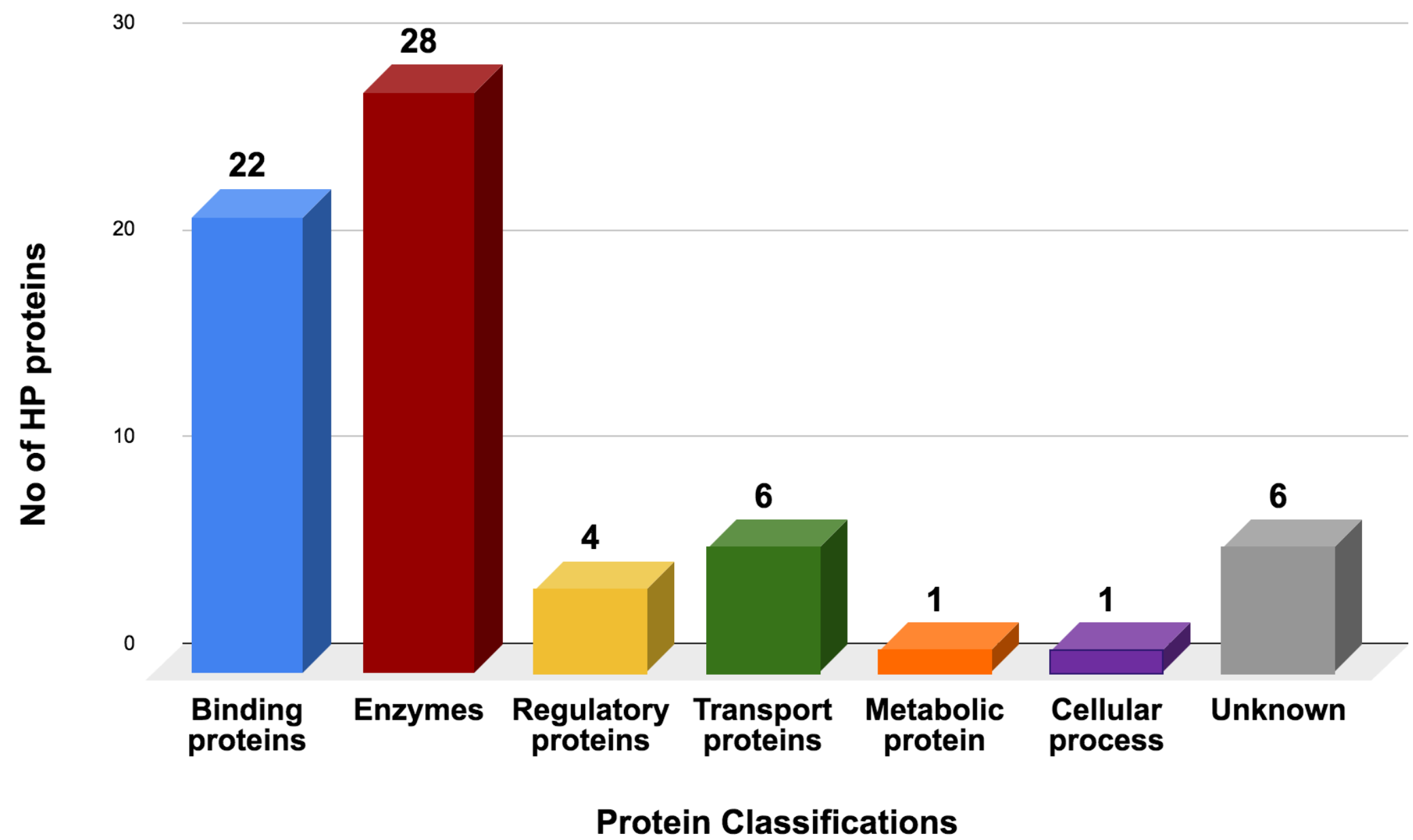


**Figure S2** Protein classifications, a total of 61 HPs out of 531 were classified into six functional protein family groups (binding proteins, enzymes, regulatory proteins, transport proteins, metabolic protein and cellular process) based on seven online tools used; CDART, InterPro, CATCH, SUPERFAMILY, ScanProsite, SMART and CDD-Blast. The total number of HPs given per each group is illustrated on the above of each bar. Seven HPs were given two possible functions. Six of the HPs functions were unknown. The chart shows that the majority of the HPs belong into Enzymes family group with 28, binding proteins family group with 22 HPs, transport proteins group with 6 HPs, regulatory proteins with only 4 HPs and metabolic protein and cellular process with 1 HP for each.

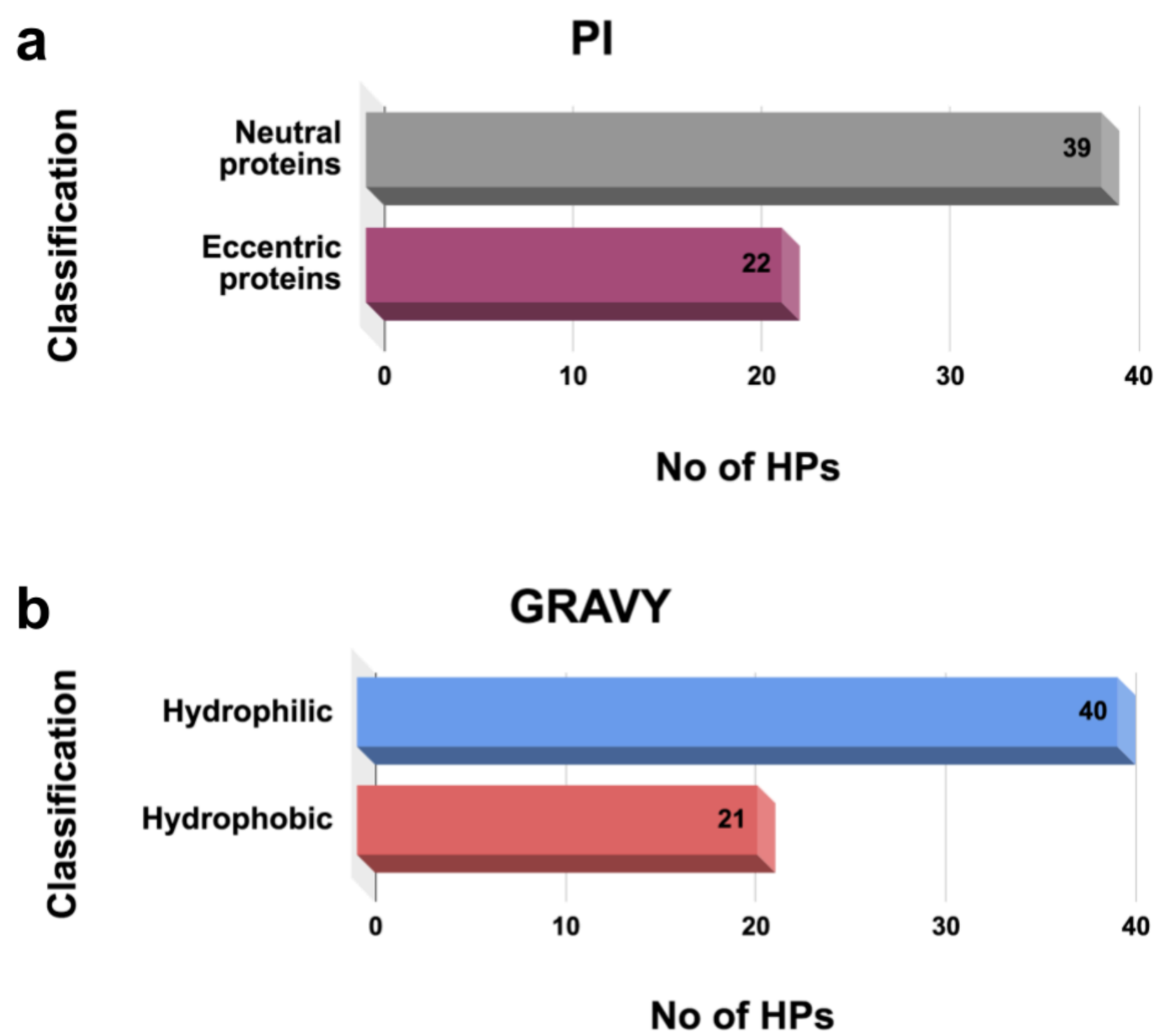


**Figure S3** Iso-electric point and GRAVY distribution among 61 HPs **(a)** Represents the iso-electric point of 61 HPs that are grouped into 39 with neutral proteins (grey bar) and 22 with eccentric proteins; 7 acidic and 15 basis (purple bar). **(b)** The bar chart shows the classification of 61 HPs based on GRAVY value in which 40 were hydrophilic(blue bar) and 21 were hydrophobic (red bar).

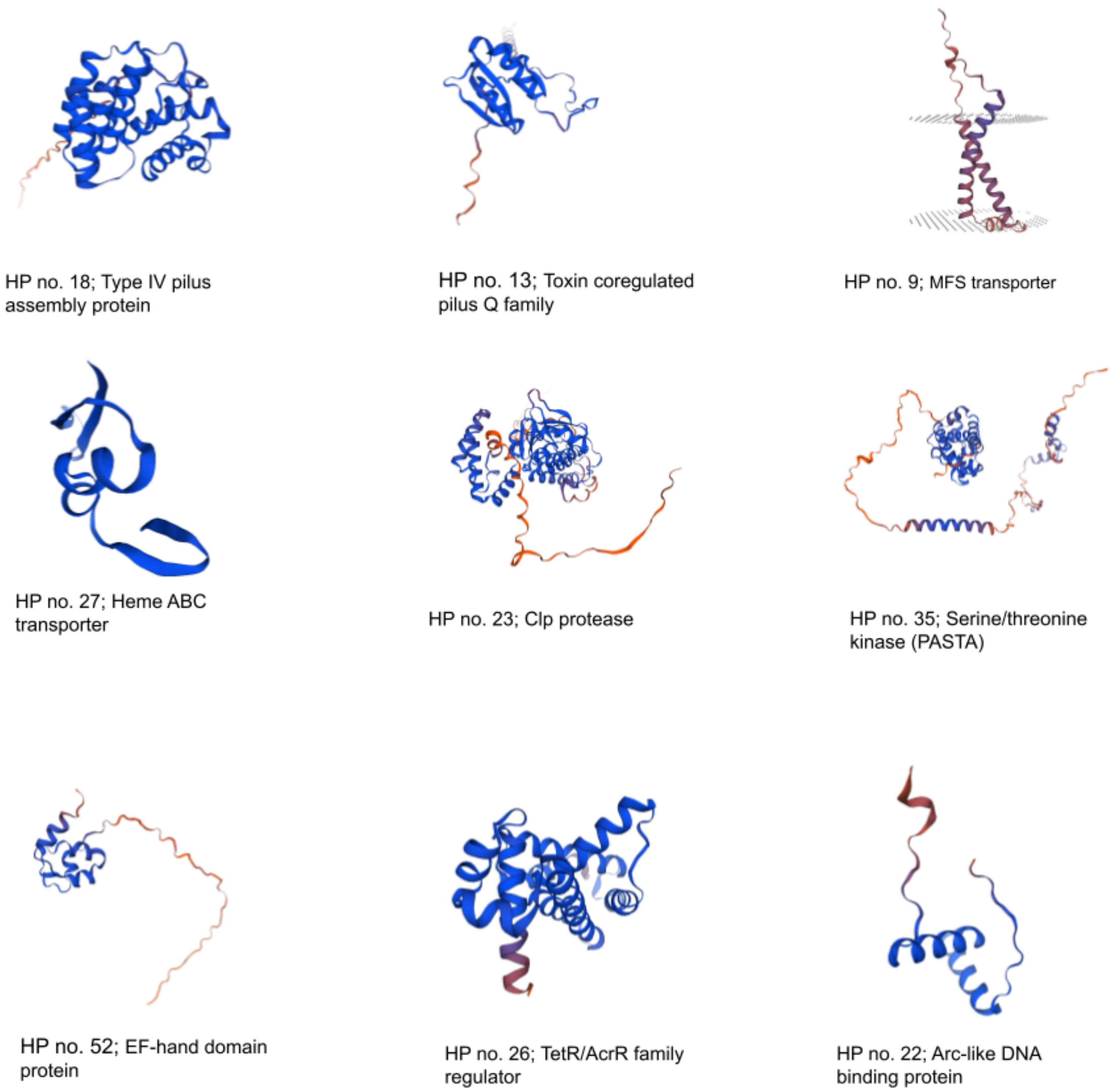


**Figure S4:** Predicted tertiary structures of the remaining nine candidate hypothetical proteins. Ribbon representations of 3D homology models generated using SWISS-MODEL. Each is individually labeled with their corresponding hypothetical protein (HP) numbers and predicted functional annotations. Secondary structural elements (**α**-helices, **β**-sheets, and loop regions) are displayed to emphasize core folding topologies. Comprehensive validation metrics—including template PDB IDs, sequence coverage (71.4%–100%), sequence identity (>89%), and GMQE confidence scores (0.65–0.93)—are provided in **Table S8**.
